# Safety of new DAAs for chronic HCV infection in a real life experience: role of a surveillance network based on clinician and hospital pharmacist

**DOI:** 10.1186/s13027-017-0119-8

**Published:** 2017-02-07

**Authors:** A. Nappi, A. Perrella, P. Bellopede, A. Lanza, A. Izzi, M. Spatarella, C. Sbreglia

**Affiliations:** 1 Pharmacy Unit, Hospital D. Cotugno – AORN Azienda dei Colli, Naples, Italy; 2 VII Division Infectious Disease and Immunology, Hospital D. Cotugno – AORN Azienda dei Colli, Naples, Italy; 3 I Division Infectious Disease, Hospital D. Cotugno – AORN Azienda dei Colli, Naples, Italy; 4 Hepatology Unit, Hospital A. Cardarelli, Naples, Italy

**Keywords:** HCV, DAAs, Antiviral, Adverse drug reactions, Pharmacology, Hepatitis C, SVR

## Abstract

**Background:**

Direct Antiviral Agents (DAAs) for HCV therapy represents a step ahead in the cure of chronic hepatitis C. Notwithstanding the promising results in several clinical trials, few data are available on adverse effects in real life settings.

**Methods:**

We have evaluated 170 patients with persistent infection and on those eligible to treatment we have followed up them through a network managed by clinician and hospital pharmacist.

**Results:**

According to our data we have found that 41% (32 out of 78) of enrolled patients experienced adverse reactions, of these 40% were in those under 65 years while 60% was in patients older than 65 years, SVR was achieved in 88% of the patients (including drop-out). We had 4 drop-out treatment due to major adverse reaction (heart and lung related).

**Conclusion:**

Even if new antiviral drugs seem to be promising, according to SVR, they require careful follow-up, possibly managed by clinician and hospital pharmacist, to avoid unrecognized side effects which may affect adherence and the real impact of these drugs on chronically infected subjects.

## Background

Hepatitis C virus (HCV) chronically infects approximately 185 million people worldwide and it still represents and important issue in public health. The rate of persistent infection after acute hepatitis ranges from 20 to 40% [1–3]. Once chronically infected patients may undergo antiviral treatment, however in the last decades, according to old antiviral regimen, chronic infection was characterized by low sustained virological response (SVR) [4]. Persistent infection can lead to cirrhosis, liver cancer, and death, and is one of the leading cause of liver transplantation in the European Country [5]. Italy has one of the highest HCV prevalence and according to data managed until 2002 with substantial geographic differences in the prevalence, with a range from 2.6% in the north [5, 6] to 16.2% in the south of Italy, However other reports suggest a decreasing trend in our country [6, 7].

Despite the lack of recent data, HCV chronic infection still remains an issue in our country. Currently thanks to the direct-acting antivirals (DAA) HCV is treatable and the goal of treatment is to achieve a sustained virological response (SVR), considered to be a functional cure (absence of plasma HCV RNA 12 weeks after completing therapy). [4]. In addition, these new antivirals have been demonstrated to be effective regardless of race, gender, or HIV status, leaving few barriers to treatment having so the potential to reduce long-term costs of complications and interrupt the current global HCV epidemic even if more expansive than previous regimen. [8, 9]. However several drug to drug interactions have been reported for some of these, requiring careful in the management. According to previous studies on first line antiviral as protease inhibitors SVR rates increased with the use of these drugs but so did the adverse events, resulting in discontinuation rates of 9–19% in patients on these triple therapy regimens [10]. Therefore the new DAAs seem to have all quality to be considered as a miracle drug [11]. However despite these new drugs has been presented as the new miracle in the infectious disease and been characterized by a very low adverse events rate in the published clinical trials, few data are available on adverse events based real life studies [12]. At the beginning of 2015 when DAAs were available in Campania Region in south Italy, where HCV is epidemic, we decided to assess impact of these new drugs on healthiness of the patients according to their adverse reactions. This kind of approach has been managed trough the creation of a network involving clinician and pharmacist to improve the follow-up of the patients under treatment not only from the efficacy point of view but mainly according to safety of these antivirals. Here we present our analysis and results on a surveillance network based on clinician and pharmacist to evaluate the safety of DAAs for HCV chronic infection in a real life in out-patients clinic of a tertiary care infectious disease division of a regional Hospital Center for Infectious disease in Campania Region.

## Methods

**Fig. 1:**
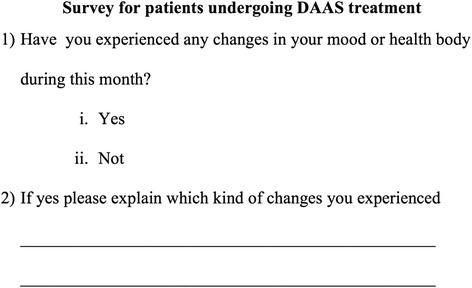
Figure shows the survey proposed by Clinician every month during treatment period. According to possible changes in health status perceived by the patient as well as any relevant clinical and laboratory condition, adverse events notification were reported and discussed with Hospital Pharmacist and entered in the online based Italian system for adverse drug reaction notifications

All patients were enrolled in this study according to national guidelines for the evaluation of HCV treatment eligibility assessed following the priority criteria established by the national registry of the Italian Medicines Agency committee (AIFA) (www.agenziafarmaco.gov.it). Data related to the efficacy of the DAAs is not the primary objective of the study therefore they are treated marginally. Data related to adverse drug reactions were collected through standard-of-care operating procedures utilized in a specialty pharmacy setting. These procedures utilized prescription claims software and a clinical assessment management program according to national network for pharmacovigilance (RNF - Rete Nazionale Farmacovigilanza). All patients were counseled prior to receiving their initial prescription according to clinician evaluation in out-patients clinic. Further, during all follow-up a survey based on two simple questions was also proposed and collected by clinician to assess the psychological health status in the course of therapy and to assess possible unrecognized side effects every month during therapy (Fig. 1). All patients were invited to communicate any changes in their health status or wellbeing during the entire treatment period. All concomitant therapies were evaluated and possible drug to drug interactions were assessed according to producer package insert and University of Liverpool web site (http://www.hep-druginteractions.org/). Patients were encouraged to contact their clinicians to report any adverse reaction during treatment. Before the enrollment in any treatment regimen, every patients signed an informed consent under the prescribing physician surveillance. Any enrolled patients performed the following laboratory tests at the following time points: T0 (before starting treatment genotype, initial viral load, HBsAg, Anti-HIV, Haematological, Liver, Renal, Pancreatic Function Test, Cardiological assessment (including Pro-BNP serum levels), at T1 and T3 according to antiviral schedule (on month and three months after starting therapy) Viral load, ETR (the end of treatment) and one month and three months after the end of therapy to evaluate sustained virological response (SVR). Red blood cells count and haemoglobin levels were assessed every week for the first month thereafter every two weeks or according to haematological alterations. Every patient underwent a clinical examination in out-patients clinic and any significant clinical condition was registered and used in case of treatment suspension or antiviral dose adjustment. Adverse events were defined according to FDA regulation (http://www.fda.gov/Safety/MedWatch/HowToReport/ucm053087.htm). All required information for possible adverse drug reaction (ADR) were entered into the RNF home page according to italian surveillance submission form (http://www.agenziafarmaco.gov.it/sites/default/files/tipo_filecb84.pdf). DAAs Regimen according to genotype and Italian National Health system guidelines were as follows: Sofosbuvir + Ribavirin, Sofosbuvir + Simeprevir +/− Ribavirin, Sofosbuvir + Daclatasvir +/− Ribavirin, Ledipasvir + Sofosbuvir +/− Ribavirin and Ombitasvir/Paritaprevir/Ritonavir/Dasabuvir plus ribavirin. Data were extracted and analyzed using Microsoft Excel and GraphPad for Mac Os X.

## Results

**Fig. 2:**
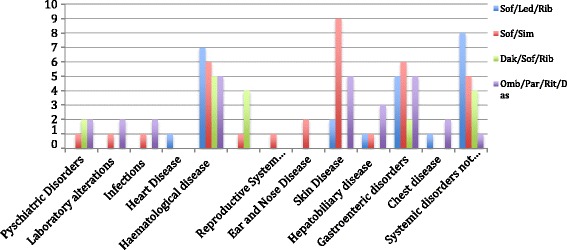
Figure represents the frequency (expressed as absolute number) of all adverse drug reactions reported in all patients during treatment follow-up. They are classified according to organ for each single antiviral schedule. According to our findings we had that patients undergoing schedule Simeprevir/Sofosbuvir had a higher frequency of skin disorders, while anaemia and asthenia were most frequently observed in those undergoing Sofosbuvir/Ledipasvir/Ribavirin treatment. Of note the Central Nervous System adverse events related to mood alteration and sleep disorders in those patients under Daklinza/Sofosbuvir antiviral schedule

**Fig. 3:**
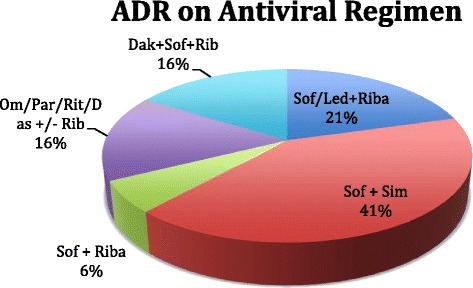
Figure represents percentage of Total Adverse Drug Reactions on the whole treated patients according to antiviral regimens. Detailed data on absolute number can be found in Table. Sofosbuvir plus Simeprevir was the regimen with more frequently reported common ADR

A total of 170 subjects were evaluated from March 2015 to March 2016. 104 out 170 were found to be eligible to HCV therapy. 78 out of 104 patients (pts) were enrolled and reached the end of treatment at the moment of our analysis based on the above mentioned antiviral regimens. Sustained virological response (SVR) was reached in 88% of the enrolled patients (percentage is actually including drop-out due to severe adverse reactions with relapse). According to our enrolment protocol to follow-up ADRs, as part of the surveillance network including clinician and hospital pharmacist, we found the following results about demographic, efficacy and safety (also reported in Table 1 and Table 2): 35% of enrolled were < 65 years old while the remaining patients (65%) were ≥ 65 years old. The cohort of subjects ≥ 65 years old had a mean age of 72 years (range, 65–80 years). In the < 65 years old cohort, the mean age was 48 (range, 18–58 years), 59% (n = 17) of the subjects were male. Almost all patients (93%) with age ≥ 65, had Genotype 1b while other genotypes were as follows Genotype 2 (4%), Genotype 3 (3%). In the subjects with age < 65, 86% of the subjects had genotype 1b while the other genotypes were Genotype 2 (6%) and Genotype 3 (15%). Elderly had a higher rate of compensated diagnosed cirrhosis according to Fibroscan as F4 (82% vs. 57%). Adverse events, categorized according to the above reported schedules from FDA and AIFA (Italian Agency for Drugs Administration) were classified as severe, when requiring hospitalization or life-threatening approach or as common when averse events could be managed in out-patients clinic without hospitalization. The most important adverse events are reported in Table 1 and Fig. 2. Basically we had a total of 37 out of 78 enrolled patients (46%) reporting common adverse drug reactions related to all used drugs. Severe adverse events were 11 out 78 pts of these reported ADRs we have that severe were mostly related to Sofosbuvir/Ledipasvir treatment and were related to cardio-pulmunary system. According to our survey we found that majority of the patients experienced asthenia or fatigue were 53% of the enrolled patients, however that adverse events did not require any dose adjustment or have any impact in social life and was considered as minor adverse drug reaction. 12 out 37 adverse events were in patients <65 years old and 25 in those > 65 years old. Major adverse events, according to FDA classification, were about 80% of all reported adverse reactions and were more frequent in those older than 65 years. The majority of ADR for treated patients were found in Sofosbuvir plus Simeprevir (Fig. 3) and in all remaining having Ribavrin as concomitant antiviral, requiring in about 60% of the patients dose reduction.

**Table 1:** Adverse events experienced by patients treated with antiviral schedule regimens

	SOF/LDV	SOF/DAK	SOF/SIM+/− RBV	SOF/RBV	OMB/PTR/r/DAS **+/− RBV**
ENROLLED PTS per regimen	**20**	**15**	**28**	**4**	**11**
PTS WITH SERIOUS ADRs	**4 (20%)**	**3 (20%)**	**1 (4%)**	**1 (25%)**	**1 (9%)**
DISCONTINUATION	3	0	0	0	1
DEATHS	0	0	0	0	0
COMMON ADRs	**10 (50%)**	**6 (40%)**	**12 (43%)**	**4 (100%)**	**5 (45%)**
FATIGUE	6	5	11	4	4
HEADACHE	0	0	0	0	0
NAUSEA	0	0	0	0	0
PRURITUS	0	0	4	0	0
INSOMNIA	1	3	0	0	0
DIARRHOEA	0	0	0	0	0
ASTHENIA	7	2	11	4	3
RASH	0	0	4	0	0
IRRITABILITY	0	4	0	0	0
ANAEMIA	6	3	12	3	3
DYSPNOEA	2	0	0	0	1

*Common adverse drug reactions are not to single patients, one patient may experience more than one common adverse drug reaction. Data are expressed as absolute number plus percentage

**Table 2:** Table shows daemographic data, SVR (in months) and ADRs according to overall enrolled population, genotype (Gt) and disease stage (chirrosis and chronic hepatitis C F3 stage according to metavir)

	CHIRROSIS	CHC	TOT
ADRs	20/51	15/27	35/78
Sex	M 44 – F 22	M 14 – F 4	M 58 - F 26
Age	<65: 10	<65: 17	<65: 27
	>65:41	>65:11	>65:51
Genotype in overall population			
Gt 1	38	20	58
Gt 2	9	5	14
Gt 3	4	2	6
SVR in over all population			
SVR 3 mts	44/51	25/27	69/78
SVR 6 mts	44/51	24/27	68/78
RELAPSE	6/51	4/27	10/78

We had 4 discontinuations during treatment representing 5% of all enrolled cases, two of those were related to heart failure during Sofosbuvir/Ledipasvir plus Ribavirin therapy, one due to allergic reaction to Harvoni and the last one related to pulmonary hypertension with heart failure during Ombitasvir/Paritaprevir/Ritonavir/Dasabuvir plus ribavirin therapy. Of note we had 4 patients during Daklatasvir/Sofosbuvir therapy experiencing change in mood of mild grade, two of them requiring Psychiatric assessment, however none of those patients required therapy suspension. All psychiatric symptoms diminished and disappeared after two weeks from the end of therapy with Daklatasvir/Sofosbuvir.

## Discussion

Despite major progresses have been made in the treatment of chronic hepatitis C, patients should always be managed with caution to avoid the side effects of therapy. Currently the choice of DAAs should be made according to viral genotypes and treatment history to avoid cross-resistance issues [8, 13]. As more safety and efficacy data are becoming available in compensated cirrhosis, antiviral therapy should be considered a priority in these patients and treatment should also be started based on possible adverse reactions and therefore related clinical implications according to age and possible pre-existing factors. In this context, all relevant clinical conditions prior of antiviral treatment should also be careful evaluated being possibly correlated to the onset of adverse events during treatment. For instance, the management of decompensated cirrhotic, could result to be more difficult to manage and to be predictable in its complications as only a few studies of DAA combinations are available [9, 12]. Despite these patients should be treated in an urgent manner, on the other hand they should be managed with caution as at now only few safety data are available for DAAs in real life on the above mentioned clinical condition. Same consideration should also be done for possible cardiovascular system adverse events in those patients having cardiac diseases, since the new antivirals seem to be related to the onset of cardiotoxicity, particularly in elder population [13, 14]. In fact, according to our results on adverse events and some literature evidences [13, 14], all patients, particularly those over 65 years, should be referred to a reference centre in case of rapid clinical deterioration. The same caution applies in the pre- and post-transplant setting where drug-to-drug interactions, kidney function and many other factors should be taken into consideration [13]. In our study, we have found a high rate of a total ADR in enrolled patients compared to previous report, particularly we had higher serious adverse events in patients undergoing Sofosbuvir/Ledipasvir, Daclatasvir/Sofosbuvir compared to those reported in published clinical trials [15] particularly in those aged over 65 years. It is of note that in those latter subjects we had the most critical ADR requiring in four cases treatment suspension due to major severe adverse events, life threatening, related to heart function (submitted papers as clinical report). Regarding common adverse events, they have been previously reported to range from 10 to 50% in several clinical trials [15]. In our study of real life we have found a percentage of about 50% with some relevant clinical condition related to mood alterations during Daclatasvir/Sofosbuvir schedule not previously reported in common adverse events at our knowledge. It also should be said that our findings on all reported adverse drug reactions could also be strictly correlated to the presence of a surveillance network based on clinician and pharmacist cooperation. Indeed, one of the most interesting results of our study is mainly the usefulness of that kind of approach to the follow-up based on a network between clinician and pharmacist. Indeed this approach may also justify the evidence of a wider and more detailed assessment of adverse reaction that patients may experience compared to other previous reported paper [12]. Further, it seems also to be really interesting and effectiveness the use of a simple survey as alerting system for clinician and therefore pharmacist for a wide understanding of possible unrecognized adverse events. Certainly, it is our opinion that without this network and that kind of approach, in a real life setting, it would be really hard to identify minor adverse events related to these antivirals that could have important impact on patients. According to previous evidences and our results on possible cardiovascular system adverse events [14–16], a well defined approach and management to this new treatment focusing on ADR according to our network may really be useful for future strategies in treatment schedule in particular setting of patients as our findings on over 65 years old seem to suggest.

## Conclusion

Therefore, in conclusion, even if DAAs seem to be promising for their ability to achieve SVR, a careful and clinician and pharmacist based network should be managed to have a better understanding and follow-up of any significant adverse reaction that may occur particularly on cardiovascular system and in elderly patients. This approach should be used in all real life study to have a wider and better approach to the use of new drugs.

## Abbreviation

ADR: Adverse drug reaction
DAAs: Direct antiviral agents
FDA: Food and drug administration
HCV: Hepatitis C virus
SVR: Sustained virological response
